# Early spring sex differences in luteinizing hormone response to gonadotropin releasing hormone in co-occurring resident and migrant dark-eyed juncos (*Junco hyemalis*)

**DOI:** 10.1016/j.ygcen.2016.06.031

**Published:** 2016-09-15

**Authors:** Timothy J. Greives, Adam M. Fudickar, Jonathan W. Atwell, Simone L. Meddle, Ellen D. Ketterson

**Affiliations:** a Department of Biological Sciences and Environmental and Conservation Sciences Program, North Dakota State University, Fargo, ND, United States; b Department of Biology, Center for the Integrative Study of Animal Behavior, Indiana University, Bloomington, IN, United States; c The Roslin Institute, The Royal (Dick) School of Veterinary Studies, The University of Edinburgh, Midlothian, EH25 9RG Scotland, UK

**Keywords:** Seasonal breeding, Phenology, Supplemental cues, Sex differences, Songbird

## Abstract

To optimally time reproduction, animals must coordinate changes in the hypothalamo-pituitary-gonadal (HPG) axis. The extent of intra-species variation in seasonal timing of reproductive function is considerable, both within and among populations. Dark-eyed junco (*Junco hyemalis*) populations are known to differ in their reproductive timing response to cues experienced in the same habitat in late winter/early spring. Specifically in juncos cohabitating on shared wintering grounds, residents initiate breeding and reproductive activity but migrants delay reproductive development and prepare to migrate before breeding. Here, we test the hypothesis that the pituitary gland acts as a ‘control point’ to modulate differential HPG axis activity across populations. We sampled free-living resident and migrant juncos on their shared over-wintering grounds in March, thus all individuals were experiencing the same environmental cues, including photoperiod. We predicted that during this critical time of transition, residents would more readily respond to repeated gonadotropin releasing hormone (GnRH) stimulation with increases in luteinizing hormone (LH), in contrast to migrants, which should delay full reproductive activity. Our data indicate that migrant females, while still on the overwintering grounds, have a reduced LH response to repeated GnRH injections compared to resident females. Male migrant and resident birds did not differ in their responsiveness to repeated GnRH. Our results suggest a sex difference in the costs of mistimed activation of the HPG axis, with female migrants being less responsive than residents females and males to repeated stimulation. Further, our data implicate a key role for the pituitary in regulating appropriate reproductive timing responses.

## Highlights

• In early spring female juncos vary in LH following repeated stimulation with GnRH.
• Resident and migrant males do not vary in LH.
• Suggests the pituitary as a critical point of control for reproductive timing.
• Sex difference suggests selection shaped responsiveness at critical time points.

## 1 Introduction

Most temperate-zone animals demonstrate seasonal bouts of breeding, timing reproduction such that rearing of young occurs during times of abundant resources (Baker, 1938, Bronson and Heideman, 1994, Lack, 1968, Perrins, 1970). Reproductive timing decisions in seasonal breeders have long been hypothesized to have important implications for fitness (Verhulst and Nilsson, 2008), with theory and data suggesting that timing of reproduction is both heritable and influenced by environmental factors (Price et al., 1988). However, the sexes may differ in the strength of selection acting on their reproductive timing decisions (Ball and Ketterson, 2008). Identifying the underlying physiological phenotypes that selection may act on linking timing decisions with reproductive success is needed; yet, to date researchers have failed to link individual variation in physiological traits (i.e. baseline gonadotropin hormone levels) with timing decisions (i.e. egg laying) in the wild (Caro et al., 2013a, Caro et al., 2013b, Schaper et al., 2012a, Schaper et al., 2012b, Williams, 2012a). To make clear connections between an individual’s physiology and ultimate timing decisions (e.g. copulation, fertilization and birth/hatching of young), novel, non-invasive techniques will be required.

To precisely and optimally time activation of reproductive physiology and behavior in relation to prevailing environmental conditions animals must seasonally alter the activity of the hypothalamo-pituitary-gonadal (HPG) axis appropriately. Gonadotropin releasing hormone (GnRH) is released in a pulsatile fashion from the hypothalamus to stimulate the release of the gonadotropins, luteinizing hormone (LH) and follicle-stimulating hormone (FSH), from the pituitary. While the pulse interval of hypothalamic GnRH release from birds *in vivo* is unknown, one study using quail found that slices of the area of the hypothalamus with GnRH, the medial basal hypothalamus and preoptic areas, generates pulses *in vitro* with intervals that average ∼21 min (Li et al., 1994). LH and FSH stimulate the gonads to induce growth and development of reproductive tissues and gametes as well as production and release of sex steroids. The mechanistic sources of variation underlying differential timing in HPG axis function and timing decisions within and among populations remain poorly understood, particularly in females (Caro, 2012, Caro et al., 2013b, Ketterson et al., 2015, Williams, 2012a). This investigation presents work aimed at identifying a physiological measure that may relate to timing decisions and can be obtained *in vivo* without sacrificing the animal. Future work will be needed to relate the measure to subsequent timing decisions.

Injecting an individual with a controlled dose of exogenous GnRH and measuring downstream activity of the HPG axis (i.e. GnRH challenge) is one tool that has been used successfully by a number of researchers to investigate relationships between reproductive physiology and other phenotypic traits (e.g. coloration, parental care) (McGlothlin et al., 2010, McGlothlin et al., 2008, Spinney et al., 2006). Previous work in birds has shown that a single dose of GnRH is capable of inducing a significant release of LH in non-breeding white crowned sparrows (*Zonotrichia leucophrys gambelii*) (Wingfield et al., 1979). However, gleaning useful discriminatory information by measuring the LH response after a single injection of GnRH is difficult if nearly all individuals respond in a similar manner. In the current study, we expand upon the concept of the GnRH challenge to assess the HPG axis by asking whether variation exists in LH output in response to repeated pulsatile GnRH stimulation. Individuals likely experience repeated release of endogenous GnRH when exposed to stimulatory environmental and internal signals, thus the response to these repeated GnRH challenges may indicate natural variation in ‘responsiveness’ of the pituitary to release LH and fully activate the HPG axis.

In this study we utilized two distinct populations of individuals of the same species, Dark-eyed juncos (*Junco hyemalis*) that are known to differ in their reproductive timing response to cues experienced in the same habitat at Mt. Lake, Virginia, USA, in late winter/early spring. Resident (*J. h. carolinensis*) and migrant (*J. h. hyemalis*) juncos share wintering habitats throughout the southern Appalachian mountains, often joining mixed flocks, hence experiencing apparently identical environmental cues into late winter/early spring. When held under common garden conditions male resident juncos increase testosterone secretion more precipitously than migrants during the month of March and have larger testes at the end of March (Fudickar et al., 2016). Further, under these field conditions, we recently reported resident males displaying elevated baseline levels of testosterone during mid-March, while migrants possessed lower testosterone levels and greater fat reserves compared with residents suggesting preparation for migration (Bauer et al., 2016). The mechanisms generating divergent physiological trajectories in individuals exposed to the same conditions are unknown. Such comparisons between distinct groups known to differ in timing in response to identical cues provide a critical opportunity to characterize physiological diversification, and they also point towards useful methods that can be employed for more fine-scaled exploration of variation within populations (Ketterson et al., 2015).

Specifically, we tested whether repeated GnRH injections (simulating episodic pulses of endogenous GnRH releases) exposes meaningful variation among resident versus migratory populations in downstream pituitary sensitivity. In addition we investigated whether there was a sex difference in the response to GnRH challenges, which might reflect sex differences in selective pressures for the determination of timing decisions such as breeding onset. We predicted that resident juncos would have marginally elevated baseline LH levels reflecting earlier seasonal activation of the HPG axis. Further, we predicted that migrants of both sexes would display an attenuated pituitary gonadotropin (LH) response following repeated simulation as compared to residents, and that migrant females would display the most substantial reductions in LH output, reflecting the sex-specific costs associated with mistimed ovarian growth and follicle maturation.

## 2 Methods

### 2.1 Study system

The Dark-eyed Junco (*J. hyemalis*), is a songbird that is broadly distributed across North America. Populations vary widely in when they breed, and in migratory tendency (Atwell et al., 2011, Nolan et al., 2002). Migratory and sedentary populations are known to co-occur during winter and early spring when they are exposed to the same environment (e.g., day length, temperature), yet differ in whether they migrate and when they breed. Here we compare resident (*J. h. carolinensis*) and migrant (*J. h. hyemalis*) dark-eyed juncos captured on their shared wintering grounds in the Appalachian mountains of western Virginia, USA. All procedures were approved by the NDSU University Institutional Animal Care and Use Committee and conducted under appropriate scientific collecting permits issued by the Virginia Department of Game and Inland Fisheries (permit #47553) and the US Fish and Wildlife Service (permit #MB093279).

### 2.2 Capture and morphological measures

Resident (male n = 22, female n = 6) and migrant juncos (Male n = 19, female n = 8) were captured on their shared wintering grounds in Virginia (March 2013), roughly 4 weeks prior to the historical first egg laid in the resident population and roughly 2–3 weeks prior to the peak departure of migrants (Nolan et al., 2002). All birds were captured passively in continuously monitored, seed-baited walk-in traps or mist nests. All birds received a unique numbered metal band, and body mass (to nearest 0.1 g) and tarsus length (to nearest 0.1 mm) were measured. Additionally, both furcular and abdominal fat stores were visually scored on a 0–5 scale with a score of 0 meaning no visible fat and 5 meaning visible fat bulging (Nolan and Ketterson, 1983, O’Neal et al., 2011).

### 2.3 Blood sampling and hormone injections

**Fig. 1:**
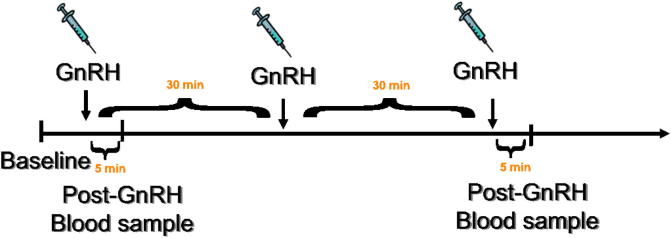
Schematic outline of sampling procedure: Briefly, immediately after capture a blood sample was collected for baseline levels of LH. Individuals then received the first injection (i.m.) with GnRH followed five minutes later with another small blood sample to measure LH. Exactly thirty minutes after the first GnRH injection, individuals received a second injection with GnRH, followed by a third thirty minutes later (60 min after the first injection). A final blood sample was collected five minutes after the third GnRH injection (65 min after the first GnRH injection).

Immediately after capture, a small ∼50 μl blood sample was obtained from the alar wing vein for baseline measures of LH. The birds then received an intramuscular injection into the pectoralis muscle of a dose of 62.5 μg chicken GnRH per kilogram body mass (American Peptide, Sunnyvale, CA) in PBS vehicle (∼50 μl injection volume); this dose is capable of fully activating the HPG axis in this species (Jawor et al., 2006). Five minutes following the first injection a second blood sample was taken from the wing vein for post-GnRH measures of LH. Previous work in this species and another songbird indicate LH levels peak 5 min following injection and significantly decline by 20 min and return to baseline 30 min post-injection (Bergeon Burns, 2012, Bergeon Burns et al., 2014, Wingfield et al., 1979). The birds then received a 2nd injection of GnRH 30 min after the 1st injection, followed by a 3rd injection 30 min following the 2nd injection. A final blood sample was collected 5 min following the 3rd injection of GnRH (for visual summary of sampling protocol see Fig. 1). All birds were held in an opaque bag between injections. Blood samples were stored on ice until later processing in the laboratory. Samples were centrifuged to separate red blood cells from plasma. Plasma was aspirated with a Hamilton syringe and placed into a microcentrifuge tube. Plasma was stored at −20 °C until shipped to The Roslin Institute, The University of Edinburgh for LH quantification.

### 2.4 Luteinizing hormone assay

To determine plasma LH, we used a micromodification of the radioimmunoassay described previously (Sharp et al., 1987). Briefly, the assay reaction volume was 60 μl, comprised of 20 μl of plasma sample or standard, 20 μl of primary rabbit LH antibody, and 20 μl of I^125^-labelled LH. The primary antibody was precipitated to separate free and bound I^125^ label using 20 μl of donkey *anti*-rabbit precipitating serum and 20 μl of non-immune rabbit serum. All samples were measured in duplicate in a single assay. The intra-assay coefficient of variation was 2.9% and the minimum detectable dose was 0.2 ng/ml. This LH radioimmunoassay has been used extensively in many avian species including dark-eyed juncos (Bergeon Burns et al., 2014, Deviche et al., 2000, Meddle et al., 2002, Wingfield et al., 2012).

### 2.5 Data analysis

All data were analyzed using the statistical software R v3.2.2 (R_Core_Team, 2011). Morphological variables were analyzed using 2-way ANOVA (resident/migrant status * sex). Baseline and GnRH-induced variation in LH levels were analyzed using a linear mixed-effects model (lmer function in the package lme4), with sex, migratory/resident status, and blood sampling point (i.e. baseline blood sample, sample following 1 GnRH injection, sample following 3 GnRH injections) as fixed effects and the individual ID as a random effect to control for repeated measures from the same individual. To probe significant effects further, we performed post-hoc pair-wise comparisons using the Holm-Bonferroni correction.

## 3 Results

### 3.1 Morphological variables

Body mass of residents and migrants were significantly different (F_1,50_ = 39.10, p < 0.001) with residents being generally heavier than migrants (Table 1). The sexes differed in body mass (F_1,50_ = 5.76, p = 0.02), with males being heavier than females (Table 1). No significant interaction between migratory/residency status and sex was revealed (p > 0.05). Similar to body mass, residents had longer tarsi than migrants (F_1,47_ = 17.95, p < 0.001) (Table 1) and males had longer tarsi than females (F_1,47_ = 8.95, p < 0.01) (Table 1). No interactive effect of migratory/residency status and sex on tarsus length was observed. A main effect of resident vs. migrant status on fat score was observed (F_1,48_ = 8.32, p < 0.01), with migrants observed to have higher levels of subcutaneous fat compared with residents (Table 1). No effect of sex or an interaction between sex and resident/migrant status on fat score was observed.

### 3.2 Luteinizing hormone levels and response to GnRH

The results of our model (Table 2) revealed a significant main effect of blood sampling time (F_2,97.9_ = 125.17, p < 0.01); post-hoc comparisons revealed elevated LH levels following a single GnRH injection compared with baseline levels in all groups (all p < 0.01), and elevated LH levels following 3 injections compared with baseline levels in all groups (all at p < 0.02). Further, a main effect of resident/migrant status was observed (F_1,51.9_ = 9.13, p < 0.01), with residents overall displaying elevated LH levels compared with migrants. Variation in how individuals respond to repeated GnRH stimulation was high, with some individuals continuing to elevate LH levels between the first and third GnRH injection, while other individuals leveled off or showed reductions in circulating LH levels between the samples collected following first and third GnRH injection (Fig. 3).

No main effect of sex was revealed (p > 0.05), and the two-way interactions of resident/migrant status and sex (p > 0.05), resident/migrant status and blood sampling time (p > 0.05), and sex and blood sampling time were not significant (p > 0.05).

**Fig. 2:**
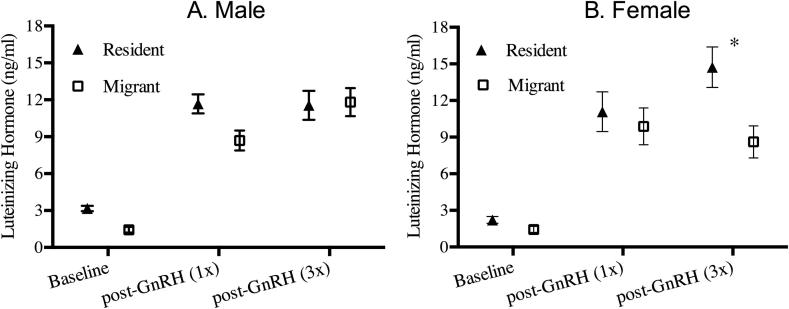
Circulating LH levels in resident and migrant juncos: A) Males: Circulating LH levels in both residents and migrants were significantly elevated following a single as well as following three GnRH injections, compared with baseline (p < 0.05). Post-hoc pair-wise comparisons revealed no significant differences between resident and migrant male juncos at any of the three sampling time points, although resident males tended to have slightly elevated levels of LH at baseline and following a single GnRH injection. B) Females: Circulating LH levels in both residents and migrants were significantly elevated following a single as well as following three GnRH injections, compared with baseline (p < 0.05). Resident and migrant female LH levels did not differ at baseline, or following a single injection with GnRH. Resident females following three GnRH injections however displayed significantly elevated LH levels compared with migrant females. * indicate significant differences revealed by post-hoc analysis (p < 0.05).

A significant three-way interaction between resident/migrant status, sex, and blood sampling time was observed (F_2,97.3_ = 4.32, p = 0.016). All 66 pair-wise comparisons derived from the 3-way interaction are reported in the Supplemental Materials. We briefly highlight here pair-wise comparisons relevant to the hypotheses we have set out to test. **Males:** Post-hoc pair-wise comparisons revealed no differences in circulating LH levels between resident and migrant males at baseline sampling (p > 0.05), following a single GnRH injection (p > 0.05) or following repeated (3x) injection with GnRH (p > 0.05) (Fig.2A). **Females:** Interestingly, while residents and migrant female LH levels did not differ at baseline (p > 0.05) or following a single GnRH injection (p > 0.05), resident females displayed elevated levels of LH compared with migrant females following three GnRH injections (t = −3.28, df, 135.05, p = 0.044) (Fig.2B).

## 4 Discussion

While much has been learned about the regulation of seasonal reproduction in male songbirds (Dawson, 2002, Dawson, 2015), the organization of the HPG axis in females leading to the breeding period is less well understood. Here we demonstrate sex and migratory status differences in the responsiveness of the pituitary to repeated GnRH stimulation. Specifically, we observed that while male migrants and residents both respond by elevating LH to similar levels when repeatedly stimulated with GnRH, resident and migrant females differ. Resident females continue to elevate LH levels in response to repeated stimulation, while migrant females fail to reach the same peak as residents, and appear to be slowly decreasing LH release in response to repeated GnRH stimulation. These data suggest that migrant females possess mechanisms capable of restraining full activation of the HPG axis to prevent erroneously timed activation of (and growth of) the ovaries.

**Fig. 3:**
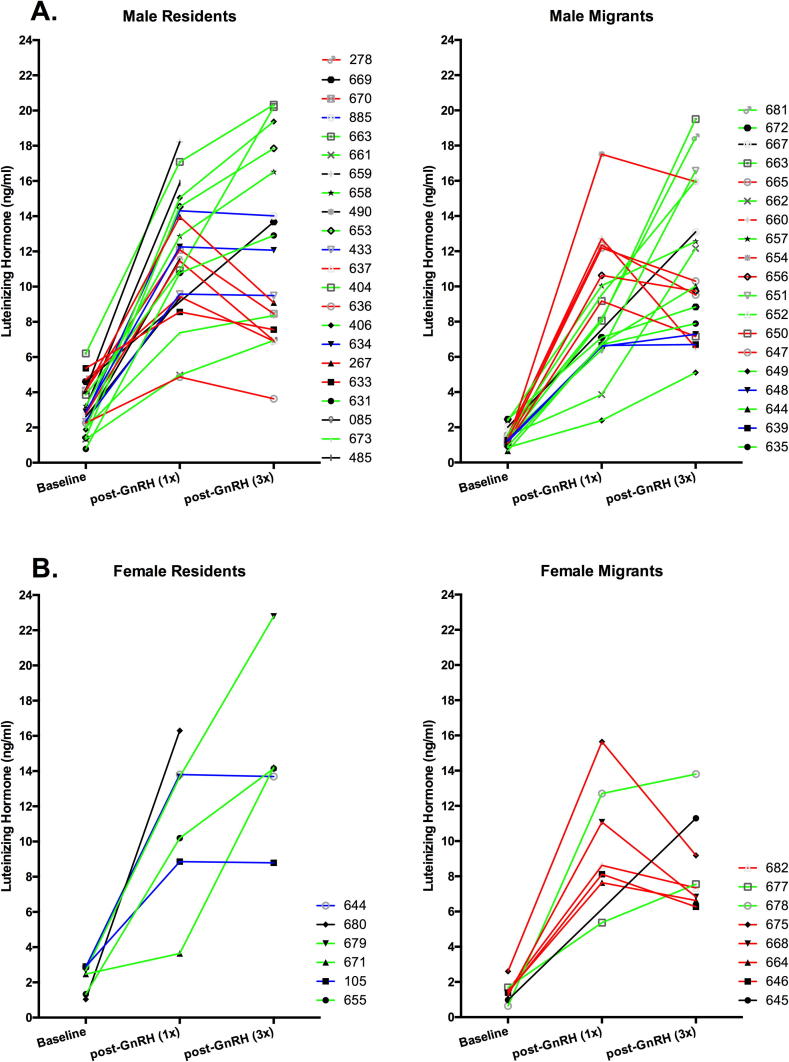
Individual response to multiple GnRH injections: Variation among individuals was observed in their response to repeated stimulation with GnRH in both males (A) and females (B). Individual responses have been provided with differing colors to aid in observation of trends, with green lines indicating continued elevation to repeated stimulation, blue lines indicating a plateau in LH levels between the 1st and 3rd injection with GnRH, and red lines indicating a drop in LH levels between initial stimulation with GnRH and repeated simulation. (For interpretation of the references to colour in this figure legend, the reader is referred to the web version of this article.)

Our data indicate that migrant females fail to reach peak LH production following repeated GnRH stimulation. Resident females, however, appear capable of responding to repeated stimulation, suggesting that perhaps they could be capable of advancing reproductive timing if presented with favorable environmental conditions (e.g. temperature (Schaper et al., 2012b). Interestingly, within resident females, there is substantial variation among individuals in their responses to this repeated stimulation (Fig. 3), suggesting the potential for similar methodology to probe for meaningful relationships within populations. Future work will investigate such relationships between this type of physiological variation and actual reproductive timing responses within resident populations where the same individual can easily be followed from the pre-breeding through the breeding season.

One likely explanation for the observed differences between HPG axis activity of resident and migrant female juncos exposed to the same conditions may be due to photoperiod-induced variation in GnRH production or release if residents and migrants possess variation in their ‘critical photoperiod’ threshold (Dawson, 2015, Silverin et al., 1993). Variation in LH stored in the pituitary may also provide a mechanism leading to the observed variation in females. Examination of the pituitary for variation in LH content at this critical time period will be needed to elucidate this possibility.

Recently we investigated the potential role that glucocorticoids may play in dampening reproductive responses in migrants as compared with residents. Previous reports in migrating songbirds suggested that birds preparing for migration should display elevated glucocorticoid levels (Holberton et al., 2007). Elevated levels of glucocorticoids have known suppressive effects on the HPG axis in other animals and contexts (Wingfield and Sapolsky, 2003). Interestingly, residents but not migrants of both sexes displayed elevated baseline and stress-induced glucocorticoid levels (Bauer et al., 2016). Further, in captive male juncos exposed to a common garden environment resident individuals also displayed elevated baseline glucocorticoid levels and increasing testosterone levels throughout the month of March (the same month sampled in the current study) (Fudickar et al., 2016). Females were not investigated in this captive common garden study. While we cannot rule out the possibility that variation in sensitivity to stress may account for the observed variation in LH response to repeated stimulation, our previous findings of reduced baseline and stress-induced glucocorticoid levels observed in migrant birds on these same study grounds (Bauer et al., 2016) suggest this is not a likely mechanism shaping observations reported here. Together, our findings suggest that glucocorticoids are not acting to inhibit activity of the HPG axis in migrants, however, whether variation exists in how glucocorticoid levels are perceived (e.g. variation in receptor abundance) remains to be elucidated.

An additional possible source of variation that might account for the observed differences in female response to repeated stimulation between residents and migrants, and among individual residents and migrants, is variation in the suppressive impact of negative feedback by sex steroids at this critical time of year. At this time, several weeks prior to clutch initiation in residents, both resident and migrant females have small ovaries that have yet to begin rapid follicular development (A. Kimmitt Personal Communication). Importantly, even small gonads can produce sex steroid secretion well in advance of full gonadal maturation and production of viable gametes (DeVries et al., 2011, Moore et al., 2002) (T. Greives and E. Stewart, unpublished observation). In songbirds, such as the songs sparrow (*Melospiza melodia*) and European starling (*Sturnus vulgaris*), sex steroids from regressed gonads of both males and females have been shown to have suppressive effects on LH secretion; removal of the gonads leads to increased LH secretion compared with intact controls, even under inhibitory day-lengths (Dawson and Goldsmith, 1984, Wilson and Follett, 1977). Steroid negative feedback at the level of the pituitary may have important implications for egg development and timing of clutch initiation. Female Japanese (*Coturnix japonica*) quail implanted with estradiol in the anterior pituitary display reduced ovulation compared with controls, likely due to a downregulation in gonadotropin secretion (Stetson, 1972). Thus, assuming the ovaries are putting out at least small amounts of sex steroids in response to GnRH in a similar manner in both residents and migrants, then enhanced negative feedback response to sex steroids at the level of the pituitary in migrants could be acting to decrease response to further GnRH stimulation in females destined to delay reproduction. Future work will investigate differences in pituitary sex steroid receptors between residents and migrants.

Our data demonstrated that migrant males, unlike females, are capable of secreting levels of LH similar to residents when presented with repeated stimulation, suggesting that, given favorable conditions or other cues, males might be capable of becoming reproductively active on this overwintering habitat, providing the possibility for gene flow if global climate change generates milder, stimulatory conditions in early spring time (Ketterson et al., 2015).

A main effect of resident/migrant status on circulating LH across all time points was observed, with residents that breed earlier in the calendar year as compared to migrants demonstrating elevated levels of circulating LH. However, following correction for multiple comparisons no difference in baseline LH levels was observed between residents and migrants of either sex. We recently reported variation in baseline activity of the male HPG axis in resident and migrant juncos, with elevated levels of testosterone in residents compared to migrants in free-living juncos caught at a similar time of year (Bauer et al., 2016). This runs counter to the lack of observable difference in baseline LH levels reported here. However, the pulsatile nature of LH (Vizcarra et al., 2015, Wilson and Sharp, 1975) may have increased variation in baseline levels to a degree that made it difficult to statistically distinguish these groups with this single measure. Thus, the main effect observed in the current study of elevated LH in residents, combined with elevated testosterone levels observed in our recent report (Bauer et al., 2016) suggests, at least in males, differential activity of the HPG axis in individuals exposed to similar photoperiodic and natural Supplementary Information. The observed reduced activity of the HPG axis in migrants compared with residents would likely lead to dampened or weaker stimulation of the gonads of birds that still must migrate back to their breeding grounds, thus reducing energy and resources directed to the gonads, and enabling allocation of resources to preparation for and execution of migration, an energetically demanding life-history stage (Wikelski et al., 2003).

The focus of the current manuscript was to explore the pituitary gland as a potential source of variation that could contribute to variation in reproductive timing responses observed in the wild (particularly in female lay-dates). Accordingly, the current study did not investigate the role of the gonad. Recent work has demonstrated that this may indeed be a critical locus of control that may also contribute to variation in timing (e.g. egg laying) (Ball, 2014, Bergeon Burns et al., 2014, Rosvall et al., 2013). Indeed, a recent report found that a desert songbird, Abert’s towhees (*Melozone aberti)*, found breeding in both natural and urban habitat displayed similar LH responses to a single GnRH injection, but testosterone secretion significantly differed between these two groups (Davies et al., 2016). Future work will be needed to address the relationship between variation in gonad responsiveness to upstream stimulation and individual variation in timing decisions.

In the current investigation, we asked if variation exists between residents and migrants and males and females in the pituitary response to exogenously applied repeated GnRH stimulation. While males of both resident and migrant groups repeatedly increased LH production in response to repeated GnRH stimulation, migrant and resident females varied in the extent of LH production and release. This sex difference likely reflects variation in selective pressures acting on mechanisms regulating seasonal reproductive timing decisions between males and females (Ball and Ketterson, 2008); females are likely to pay a greater cost of mistimed breeding than males as they must invest a significant quantity of resources into follicular maturation and egg development (Williams, 2012b).

Taken together we have demonstrated variation between the sexes in the responsiveness of the pituitary to repeated stimulation with GnRH, with males that will breed earlier (i.e. residents) and males that will breed later (i.e. migrants) capable of achieving similar LH levels, while females that will initiate their first clutch later (i.e. migrants) seemingly dampening their response to repeated stimulation compared with females that will initiate their first clutch of the season earlier (i.e. residents). Future studies aimed at relating individual variation in reproductive physiology to actual timing decisions in the field may benefit from employing the method described here which does not require sacrificing individuals.

## Figures and Tables

**Table 1:** Morphological comparisons of residents and migrant juncos (Mean ± SEM).

	Mass (g)	Tarsus (mm)	Fat Score
Resident Male	22.8 ± 0.3	22.3 ± 0.1	1.7 ± 0.1
Migrant Male	20.6 ± 0.3	21.4 ± 0.2	2.3 ± 0.1
Resident Female	21.5 ± 0.4	21.4 ± 0.3	2.2 ± 0.4
Migrant Female	20.0 ± 0.4	21.0 ± 0.3	2.5 ± 0.3

**Table 2:** Factor(s) influence on circulating LH levels: Analysis of Variance Table of type III with Satterthwaite approximation for degrees of freedom derived from our linear mixed effects model. * denotes p < 0.05, ** denotes p < 0.01, *** denotes p < 0.001. Post-hoc pair-wise comparisons can be found in Supplementary Material.

	Sum of squares	Mean square	Numerator degrees of freedom	Denominator degrees of freedom	F-value	Probability
Status (Resident/migrant)	78.6	78.6	1	51.91	9.129	0.003899^∗∗^
Sex (m/f)	0	0	1	51.91	0	0.994219
Blood sample (time point 1, 2 or 3)	2155.44	1077.72	2	97.913	125.166	<2.20E−16^∗∗∗^
Status * Sex	3.2	3.2	1	51.91	0.372	0.544655
Status * Blood sample	25.23	12.62	2	97.913	1.465	0.236032
Sex * Blood sample	3.02	1.51	2	97.913	0.175	0.839625
Status * Sex * Blood sample	74.41	37.2	2	97.913	4.321	0.015911^∗^
