# Supplementary material for: Early spring sex differences in luteinizing hormone response to gonadotropin releasing hormone in co-occurring resident and migrant dark-eyed juncos (Junco hyemalis)
Source: Gen Comp Endocrinol. 2016 Sep 15;236:17–23. doi: 10.1016/j.ygcen.2016.06.031 (PMC5036536; doi:10.1016/j.ygcen.2016.06.031)
Supplement: Supplementary data [file mmc1.docx]

Supplemental Materials:

Holm-Bonferroni post-hoc pairwise contrasts:

lsmeans

sex status Bleed lsmean SE df lower.CL upper.CL

f migrant T0 1.438750 1.1904662 130.76 -0.9163170 3.793817

m migrant T0 1.418421 0.7724765 130.76 -0.1097482 2.946590

f resident T0 2.216667 1.3746320 130.76 -0.5027304 4.936064

m resident T0 3.128636 0.7178781 130.76 1.7084775 4.548795

f migrant T5 9.943064 1.2667264 135.92 7.4380217 12.448106

m migrant T5 8.738723 0.7920878 133.02 7.1720062 10.305440

f resident T5 11.080000 1.3746320 130.76 8.3606029 13.799397

m resident T5 11.702502 0.7335335 132.71 10.2515717 13.153431

f migrant T65 8.616250 1.1904662 130.76 6.2611830 10.971317

m migrant T65 11.231579 0.7724765 130.76 9.7034097 12.759748

f resident T65 14.884808 1.4964626 137.46 11.9257431 17.843872

m resident T65 11.992493 0.7882809 137.97 10.4338192 13.551167

Confidence level used: 0.95

contrast estimate SE df t.ratio p.value

f,migrant,T0 - m,migrant,T0 0.02032895 1.4191299 130.76 0.014 1.0000

f,migrant,T0 - f,resident,T0 -0.77791667 1.8184672 130.76 -0.428 1.0000

f,migrant,T0 - m,resident,T0 -1.68988636 1.3901650 130.76 -1.216 1.0000

f,migrant,T0 - f,migrant,T5 -8.50431390 1.5296971 97.43 -5.559 <.0001

f,migrant,T0 - m,migrant,T5 -7.29997310 1.4298996 131.46 -5.105 <.0001

f,migrant,T0 - f,resident,T5 -9.64125000 1.8184672 130.76 -5.302 <.0001

f,migrant,T0 - m,resident,T5 -10.26375157 1.3983137 131.30 -7.340 <.0001

f,migrant,T0 - f,migrant,T65 -7.17750000 1.4671698 94.30 -4.892 0.0002

f,migrant,T0 - m,migrant,T65 -9.79282895 1.4191299 130.76 -6.901 <.0001

f,migrant,T0 - f,resident,T65 -13.44605765 1.9122265 135.05 -7.032 <.0001

f,migrant,T0 - m,resident,T65 -10.55374310 1.4277943 133.15 -7.392 <.0001

m,migrant,T0 - f,resident,T0 -0.79824561 1.5768110 130.76 -0.506 1.0000

m,migrant,T0 - m,resident,T0 -1.71021531 1.0545468 130.76 -1.622 1.0000

m,migrant,T0 - f,migrant,T5 -8.52464284 1.4836832 134.60 -5.746 <.0001

m,migrant,T0 - m,migrant,T5 -7.32030205 0.9680060 95.57 -7.562 <.0001

m,migrant,T0 - f,resident,T5 -9.66157895 1.5768110 130.76 -6.127 <.0001

m,migrant,T0 - m,resident,T5 -10.28408052 1.0652659 131.70 -9.654 <.0001

m,migrant,T0 - f,migrant,T65 -7.19782895 1.4191299 130.76 -5.072 0.0001

m,migrant,T0 - m,migrant,T65 -9.81315789 0.9520255 94.30 -10.308 <.0001

m,migrant,T0 - f,resident,T65 -13.46638660 1.6840785 136.18 -7.996 <.0001

m,migrant,T0 - m,resident,T65 -10.57407205 1.1036787 134.67 -9.581 <.0001

f,resident,T0 - m,resident,T0 -0.91196970 1.5507940 130.76 -0.588 1.0000

f,resident,T0 - f,migrant,T5 -7.72639723 1.8692803 133.23 -4.133 0.0024

f,resident,T0 - m,migrant,T5 -6.52205644 1.5865107 131.33 -4.111 0.0026

f,resident,T0 - f,resident,T5 -8.86333333 1.6941417 94.30 -5.232 <.0001

f,resident,T0 - m,resident,T5 -9.48583491 1.5581029 131.20 -6.088 <.0001

f,resident,T0 - f,migrant,T65 -6.39958333 1.8184672 130.76 -3.519 0.0203

f,resident,T0 - m,migrant,T65 -9.01491228 1.5768110 130.76 -5.717 <.0001

f,resident,T0 - f,resident,T65 -12.66814099 1.7944089 98.57 -7.060 <.0001

f,resident,T0 - m,resident,T65 -9.77582643 1.5846135 132.71 -6.169 <.0001

m,resident,T0 - f,migrant,T5 -6.81442753 1.4560030 134.74 -4.680 0.0003

m,resident,T0 - m,migrant,T5 -5.61008674 1.0689958 132.01 -5.248 <.0001

m,resident,T0 - f,resident,T5 -7.95136364 1.5507940 130.76 -5.127 <.0001

m,resident,T0 - m,resident,T5 -8.57386521 0.8974861 95.40 -9.553 <.0001

m,resident,T0 - f,migrant,T65 -5.48761364 1.3901650 130.76 -3.947 0.0046

m,resident,T0 - m,migrant,T65 -8.10294258 1.0545468 130.76 -7.684 <.0001

m,resident,T0 - f,resident,T65 -11.75617129 1.6597438 136.33 -7.083 <.0001

m,resident,T0 - m,resident,T65 -8.86385673 0.9427602 99.01 -9.402 <.0001

f,migrant,T5 - m,migrant,T5 1.20434079 1.4939876 135.13 0.806 1.0000

f,migrant,T5 - f,resident,T5 -1.13693610 1.8692803 133.23 -0.608 1.0000

f,migrant,T5 - m,resident,T5 -1.75943768 1.4637853 135.15 -1.202 1.0000

f,migrant,T5 - f,migrant,T65 1.32681390 1.5296971 97.43 0.867 1.0000

f,migrant,T5 - m,migrant,T65 -1.28851505 1.4836832 134.60 -0.868 1.0000

f,migrant,T5 - f,resident,T65 -4.94174376 1.9606112 136.83 -2.521 0.3731

f,migrant,T5 - m,resident,T65 -2.04942920 1.4919728 136.51 -1.374 1.0000

m,migrant,T5 - f,resident,T5 -2.34127690 1.5865107 131.33 -1.476 1.0000

m,migrant,T5 - m,resident,T5 -2.96377847 1.0795714 132.88 -2.745 0.2134

m,migrant,T5 - f,migrant,T65 0.12247310 1.4298996 131.46 0.086 1.0000

m,migrant,T5 - m,migrant,T65 -2.49285584 0.9680060 95.57 -2.575 0.3465

m,migrant,T5 - f,resident,T65 -6.14608455 1.6931637 136.55 -3.630 0.0140

m,migrant,T5 - m,resident,T65 -3.25376999 1.1174927 135.61 -2.912 0.1345

f,resident,T5 - m,resident,T5 -0.62250157 1.5581029 131.20 -0.400 1.0000

f,resident,T5 - f,migrant,T65 2.46375000 1.8184672 130.76 1.355 1.0000

f,resident,T5 - m,migrant,T65 -0.15157895 1.5768110 130.76 -0.096 1.0000

f,resident,T5 - f,resident,T65 -3.80480765 1.7944089 98.57 -2.120 0.9121

f,resident,T5 - m,resident,T65 -0.91249310 1.5846135 132.71 -0.576 1.0000

m,resident,T5 - f,migrant,T65 3.08625157 1.3983137 131.30 2.207 0.7842

m,resident,T5 - m,migrant,T65 0.47092263 1.0652659 131.70 0.442 1.0000

m,resident,T5 - f,resident,T65 -3.18230608 1.6665749 136.61 -1.909 1.0000

m,resident,T5 - m,resident,T65 -0.28999152 0.9558555 100.50 -0.303 1.0000

f,migrant,T65 - m,migrant,T65 -2.61532895 1.4191299 130.76 -1.843 1.0000

f,migrant,T65 - f,resident,T65 -6.26855765 1.9122265 135.05 -3.278 0.0438

f,migrant,T65 - m,resident,T65 -3.37624310 1.4277943 133.15 -2.365 0.5457

m,migrant,T65 - f,resident,T65 -3.65322871 1.6840785 136.18 -2.169 0.8267

m,migrant,T65 - m,resident,T65 -0.76091415 1.1036787 134.67 -0.689 1.0000

f,resident,T65 - m,resident,T65 2.89231456 1.6913862 137.57 1.710 1.0000

P value adjustment: holm method for 66 tests
